# Supplementary material for: Chromatin modifier MTA1 regulates mitotic transition and tumorigenesis by orchestrating mitotic mRNA processing
Source: Nat Commun. 2020 Sep 8;11:4455. doi: 10.1038/s41467-020-18259-1 (PMC7479136; doi:10.1038/s41467-020-18259-1)
Supplement: Supplementary file 1 — Supplementary Information [file 41467_2020_18259_MOESM1_ESM.pdf]

SUPPLEMENTARY INFORMATION

Chromatin modifier MTA1 regulates mitotic transition and tumorigenesis by orchestrating mitotic mRNA processing

Liu et al.

Supplementary Figures

Supplementary Figure 1

Supplementary Figure 2

Supplementary Figure 3

Supplementary Figure 4

Supplementary Figure 5

Supplementary Figure 6

Supplementary Figure 7

Supplementary Figure 8

Supplementary Figure 9

Supplementary Figure 10

Supplementary Tables

Supplementary Table 1

Supplementary Table 2

Supplementary Table 3

Supplementary Table 4

Supplementary Figure 1

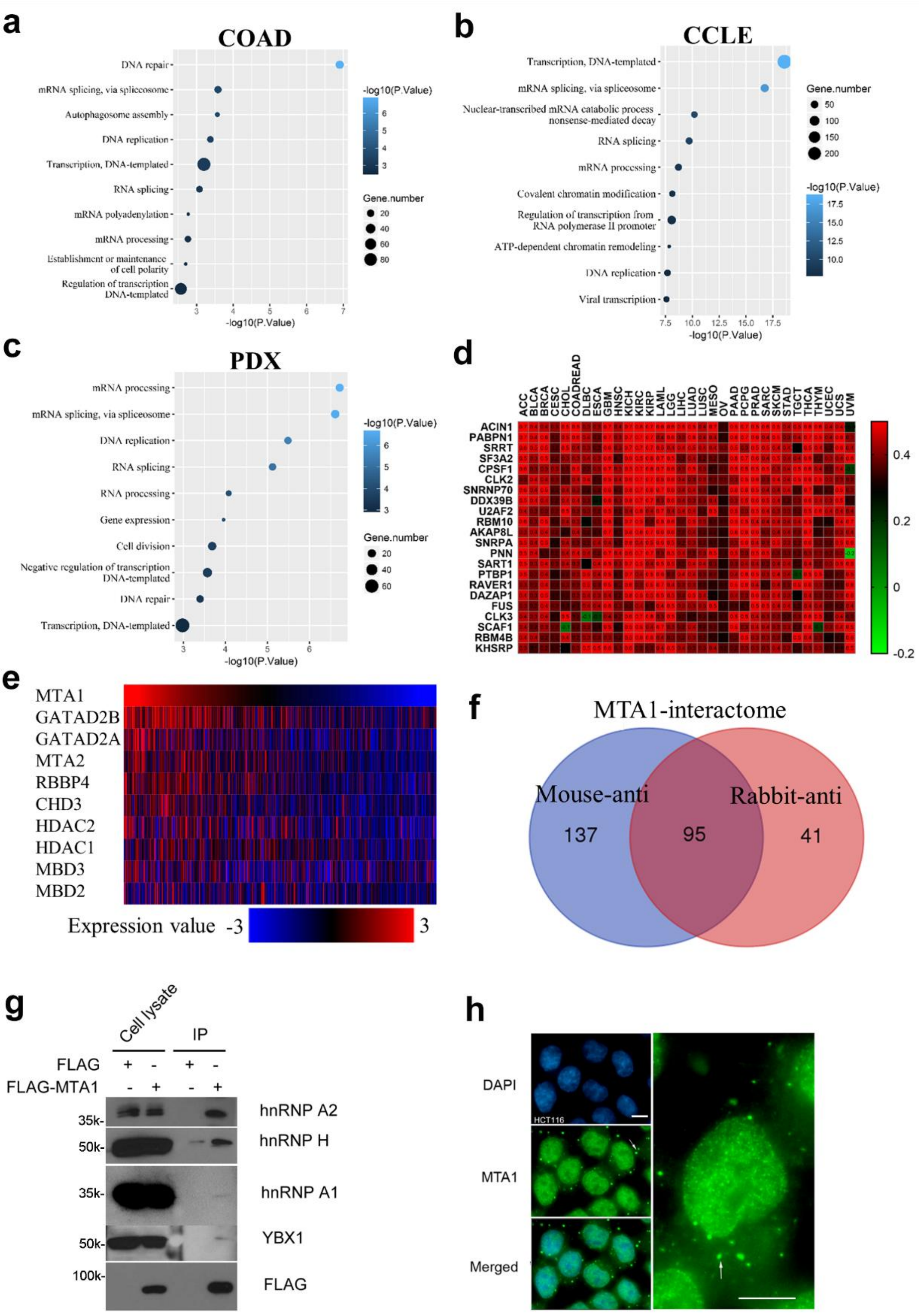

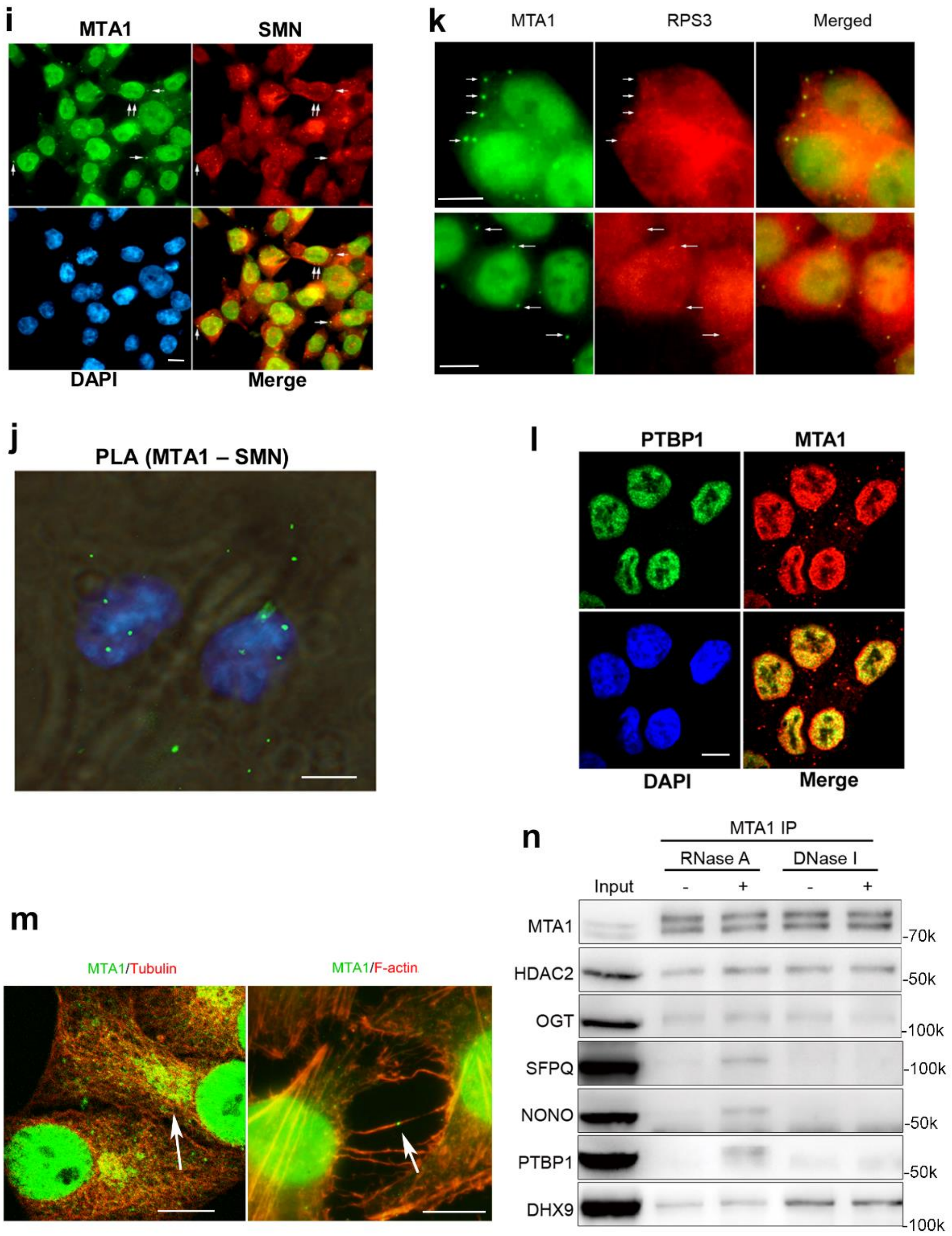

Supplementary Fig. 1 MTA1 is globally coexpressed and interacts with RBPs in cancer. **a-c** Functional enrichment analysis of the *MTA1*-coexpressed genes from the TCGA COAD, CCLE or one PDX dataset, respectively. **d** Some of reprehensive RBPs that coexpressed with MTA1 across the 32 types of cancer in the TCGA database. **e** Heatmap shows the coexpression relationship between MTA1 mRNA and transcripts of its interactors in NuRD complex in the CCLE dataset. *GATAD2A* (r= 0.346, P<0.0001), *GATAD2B* (r= 0.4075, P<0.0001), *HDAC1*(r= 0.2026, P<0.0001), *HDAC2* (r= 0.2677, P<0.0001), *MBD3* (r= 0.1521, P<0.0001), *MBD2* (r= 0.09577, P=0.0029), *MTA2* (r= 0.346,

P<0.0001), *RBBP4* (r= 0.3754, P<0.0001), *CHD3* (r= 0.3061, P<0.0001) and *CHD4* (r= 0.3556, P<0.0001). **f** Overlap analysis of the MTA1 interactors that were pulled down by the two antibodies. p-value = 5.52e-159, hypergeometric test. **g** In-vitro Co-IP experiments in HCT116 cells using the flag antibody showing the interaction between exogenous MTA1-flag and four classical RBPs. **h** Immunofluorescence experiments showing the granule-like distribution of MTA1 (green) in the cytoplasm of the HCT116 cells. **i** Immunofluorescent colocalization analysis of MTA1 (green) and SMN (red) without UV irradiation treatment. Only a proportion of the two types of granules are co-localized under normal circumstances. **j** In situ proximity ligation assay detection of the interaction between MTA1 and SMN without UV irradiation treatment. **k** Immunofluorescent colocalization analysis of MTA1 (green) and RPS3 (red). **l** Immunofluorescent colocalization analysis of MTA1 (red) and PTBP1 (green). **m** Immunofluorescence analyses showing the distribution of the MTA1 (green) cytoplasmic granules along the microtubule and microfilament cytoskeletons. Red: Tubulin in left part; Red: F-actin in right part. **n** Co-IP experiments under different conditions to determine the dependence of the MTA1-integrated protein complex on RNA or DNA. The cells were treated with RNase A and DNase I for the experiment, respectively. Modified one-tailed Fisher's exact test was used to calculate *p* values by DAVID in **a-c.** Results in **g-n** are representative of three independent repeats with similar results. Scale bar = 10 µm for **h-m**.

Supplementary Figure 2

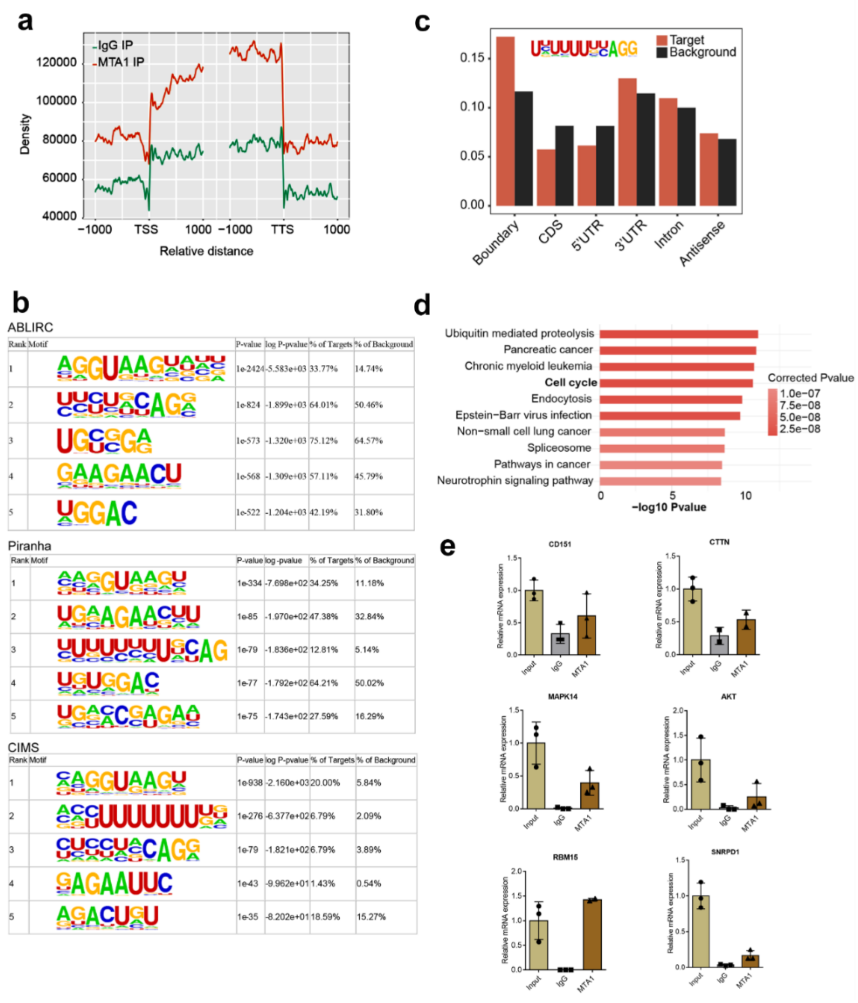

Supplementary Fig. 2 MTA1 complex binds plenty of pre- and mature RNAs. a Read distributions around the TSS and TTS showing the enriched and consistent high level in the transcriptional regions for the MTA1 binding signal. **b** Motif analysis result from three independent peak-calling software ABLIRC, Piranha and CIMS showing the consistent binding motif of MTA1. **c** The frequencies of the 3’ splice site U-rich motif in the peaks separated by regions. **d** Bar plot showing the enriched KEGG pathways for the MTA1 complex bound mRNAs. **e** Bar plot showing the RIP-qPCR validation for the MTA1 complex bound transcripts. Input and IgG were treated as controls. Data are represented as mean ± SD (n = 3 per group except CTTN IgG n = 2, RBM15 MTA1 n = 2).

Supplementary Figure 3

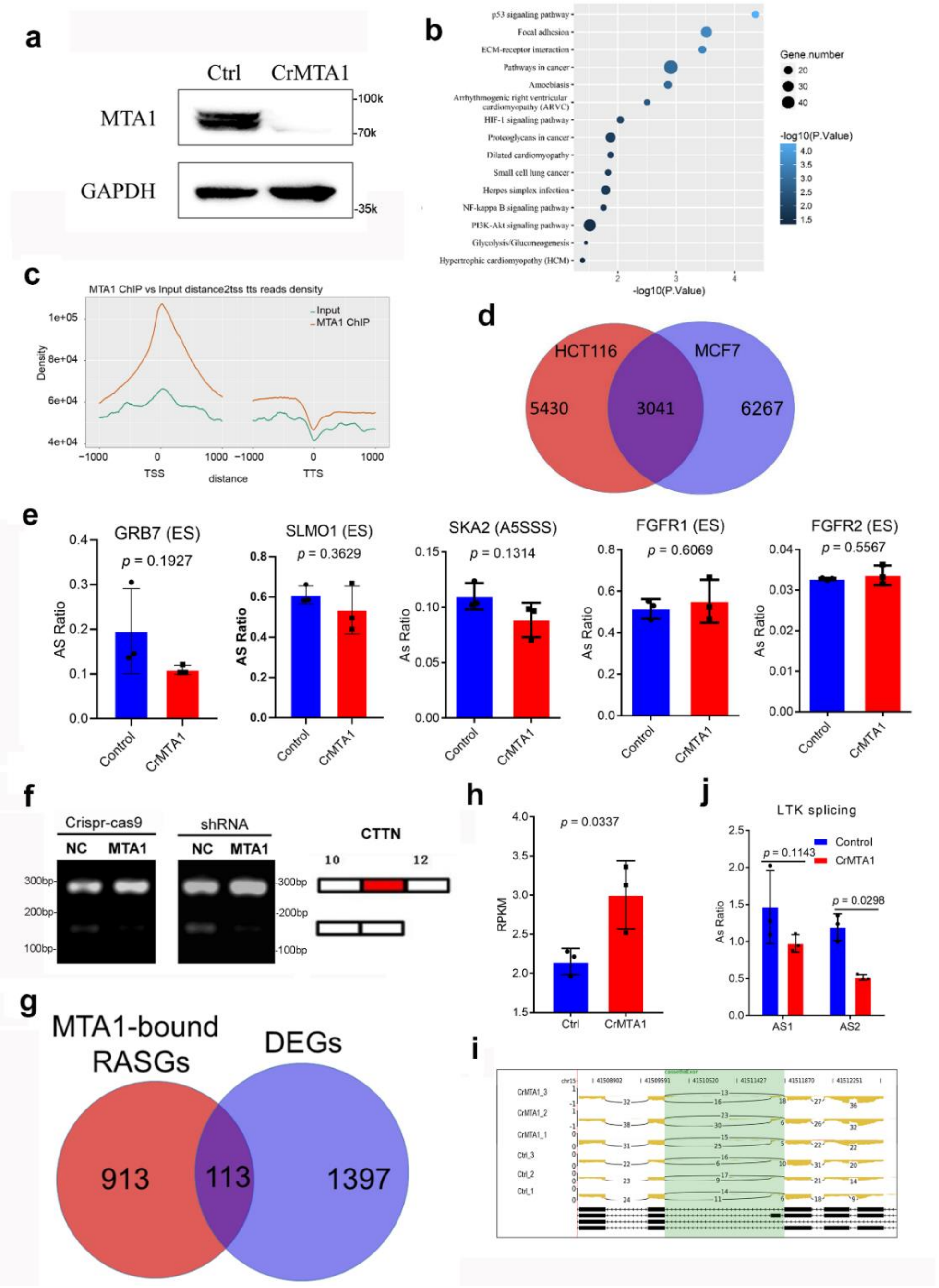

Supplementary Fig. 3 MTA1 influences transcript abundance and AS. a Western blot showing that MTA1 was knocked out by CRISPR-Cas9 technology. **b** KEGG pathway analysis of the DEGs. P value was calculated using modified one-tailed Fisher's exact test by DAVID. **c** Read distributions around the TSS and TTS site for the MTA1 ChIP and Input samples. Peaks around the TSS emerged for the MTA1 ChIP sample. **d** MTA1 ChIP-seq

data (GSE91687) from breast cancer cell lines (MCF-7) generated by ENCODE project was downloaded and analyzed. Venn diagram showing a high overlap of the binding genes between the ChIP-seq studies using the HCT116 and MCF-7 cells. **e** RT-qPCR validation for MTA1 deletion–induced splicing events. **f** RT-PCR detection of the AS change of *CTTN* after MTA1 knockdown by crispr-cas9 or shRNA. **g** Venn diagram showing the overlap between the MTA1-bound RASGs and DEGs. **h** Bar plot showing the increased transcription level alternation of *LTK* after MTA1 deletion. **i** Reads density illustration showing the exon skipping events regulated by MTA1. **j** RT-qPCR validation for the ES events in *LTK*. The alternative exon was retained to a greater degree after the MTA1 deletion. **a, f** Representative of three independent repeats with similar results. **e, h** Two-tailed Student's t-test. **j** Two-Way ANOVA with Bonferroni post-test correction. All error bars represent mean ± SD (n = 3 per group).

Supplementary Figure 4

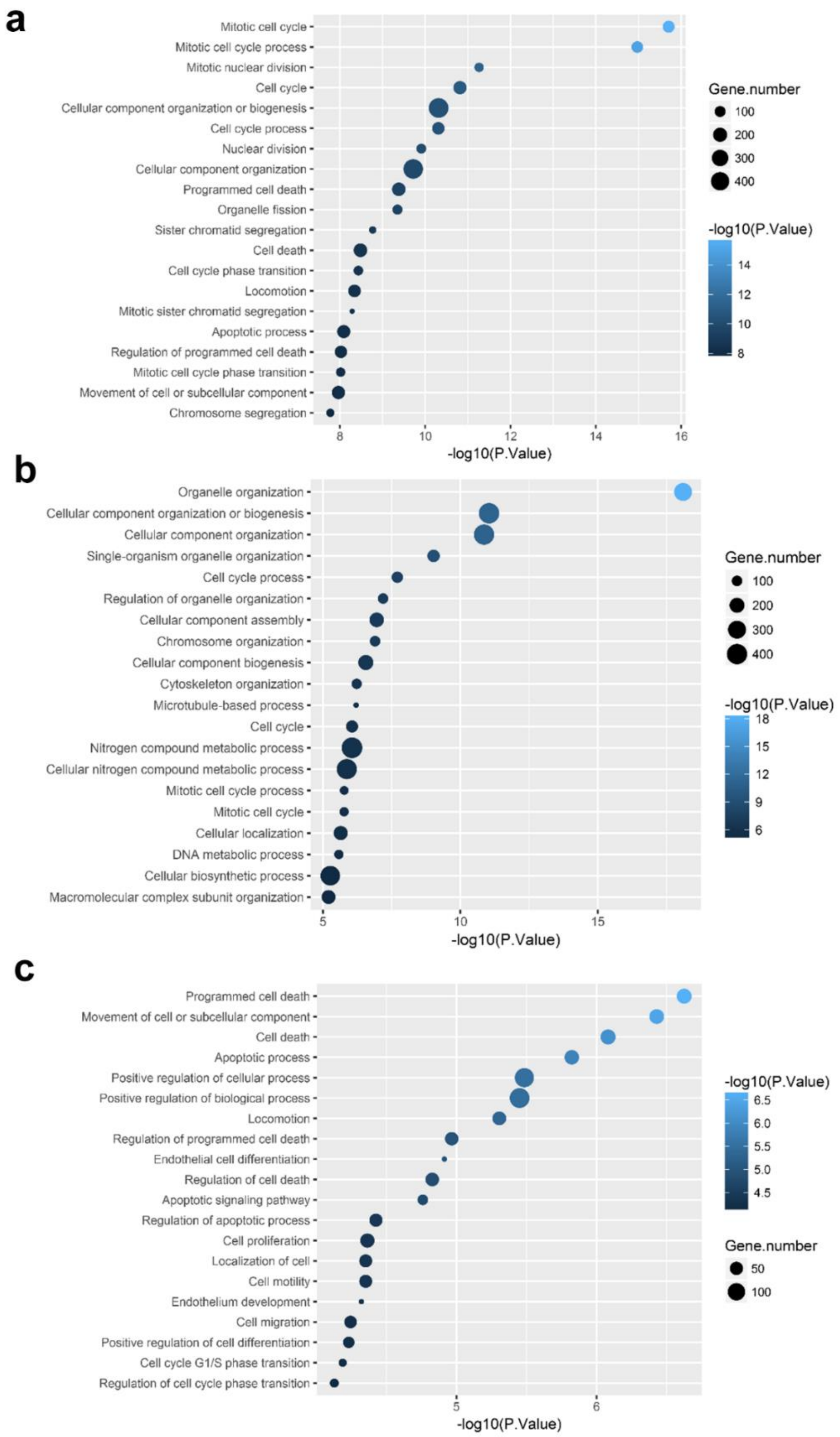

d

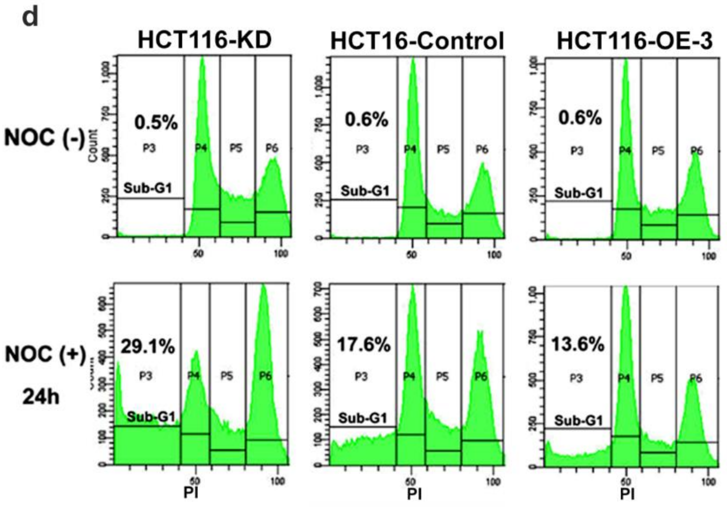

e

NOC (+) 24h

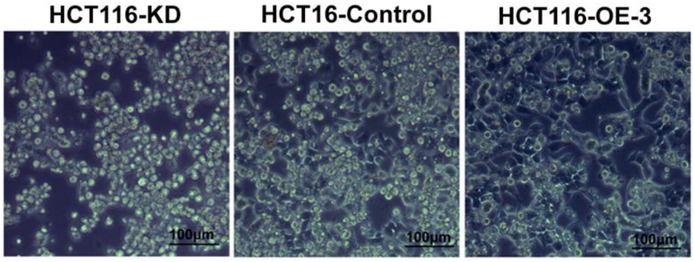

Supplementary Fig. 4 MTA1 regulates mitotic transition of cancer cells. a-c Bubble diagrams showing the functional enrichment results of the MTA1-bound DEGs, MTA1-bound RASGs and promoter-binding DEGs, respectively. The top 20 items are shown. Modified one-tailed Fisher's exact test was used to calculate *p* values by DAVID. **d** Flow cytometry measurement of the mitotic arrest-induced cell death (sub-G1) proportion after treatment with nocodazole for 24 h. **e** Observation of the changes in the cell morphology under microscopy after the cells were treated with nocodazole for 24 h. Shown is representative of three independent repeats with similar results.

Supplementary Figure 5

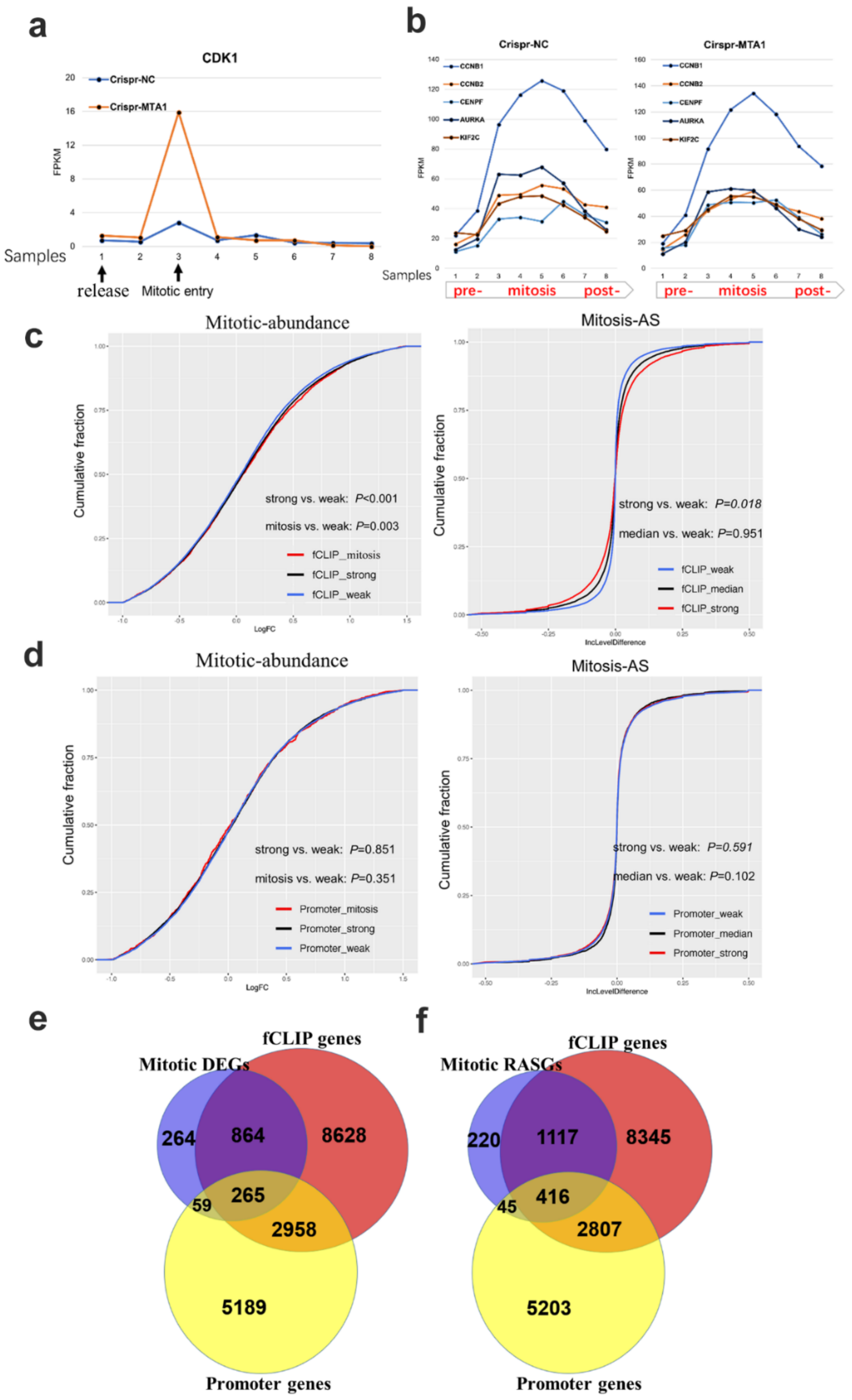

Supplementary Fig. 5 MTA1 posttranscriptionally governs the mitotic transcriptome. **a** The synchronized cell lines by double-thymidine block entered mitosis at the third time point according to the expression of the mitotic entry marker CDK1. **b** The sequentially

synchronized cell samples were further classified into premitosis, mitosis and postmitosis subgroups according to the expression of mitosis specific markers, including CCNB1, CCNB2, CENPF, AURKA and KIF2C. **c** The fCLIP target transcripts were classified as strong- or weak-affinity with MTA1, and the empirical cumulative distribution function of the log2 CrMTA1/Control fold changes of the detected abundance (left) or AS (right) for each set in synchronized mitotic samples were plotted. The bound mitosis-related gene transcripts were also plotted. **d** The CHIP promoters were classified as strong-, median- or weak-affinity with MTA1. The empirical cumulative distribution function of the log2 CrMTA1/Control fold changes of the abundance (left) and AS (right) for each set in synchronized mitotic samples were plotted. **e** Venn diagram showing the overlapping genes among the MTA1 deletion-induced DEGs in the mitotic samples, the MTA1 fCLIP-genes in the fCLIP-seq data and the MTA1 promoter-binding genes in the CHIP-seq data. **f** Venn diagram showing the overlapping genes among the MTA1 deletion-induced RASGs in the mitotic samples, the MTA1 fCLIP-genes in the fCLIP-seq data and the MTA1 promoter-binding genes in the ChIP-seq data. **c-d** One-way ANOVA with Tukey's multiple comparisons test.

Supplementary Figure 6

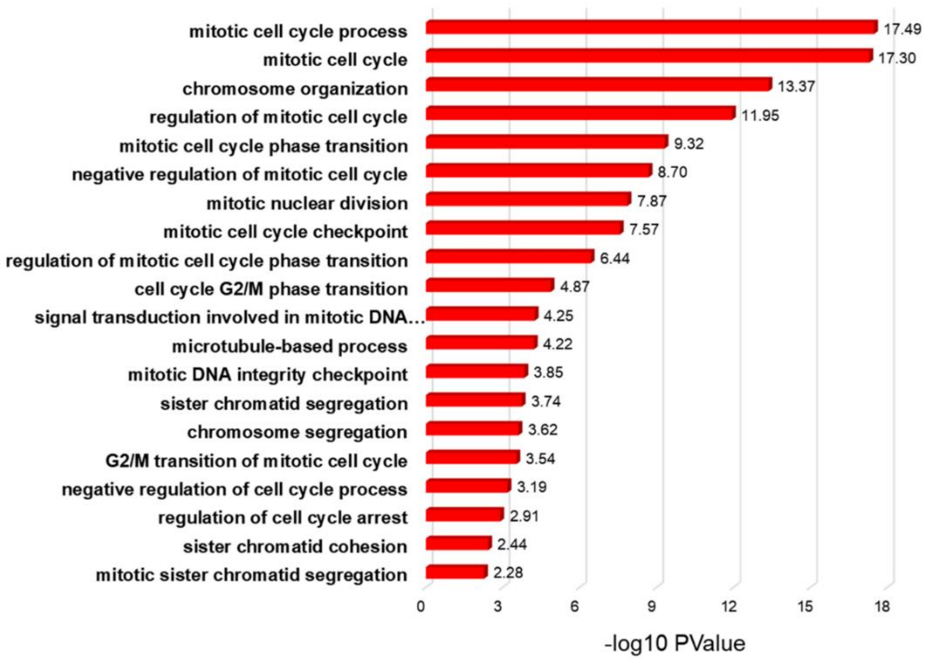

Supplementary Fig. 6 MTA1 deletion-induced DEGs are enriched on mitosis. Only the mitosis-related items are shown. Modified one-tailed Fisher's exact test was used to calculate p values by DAVID.

Supplementary Figure 7

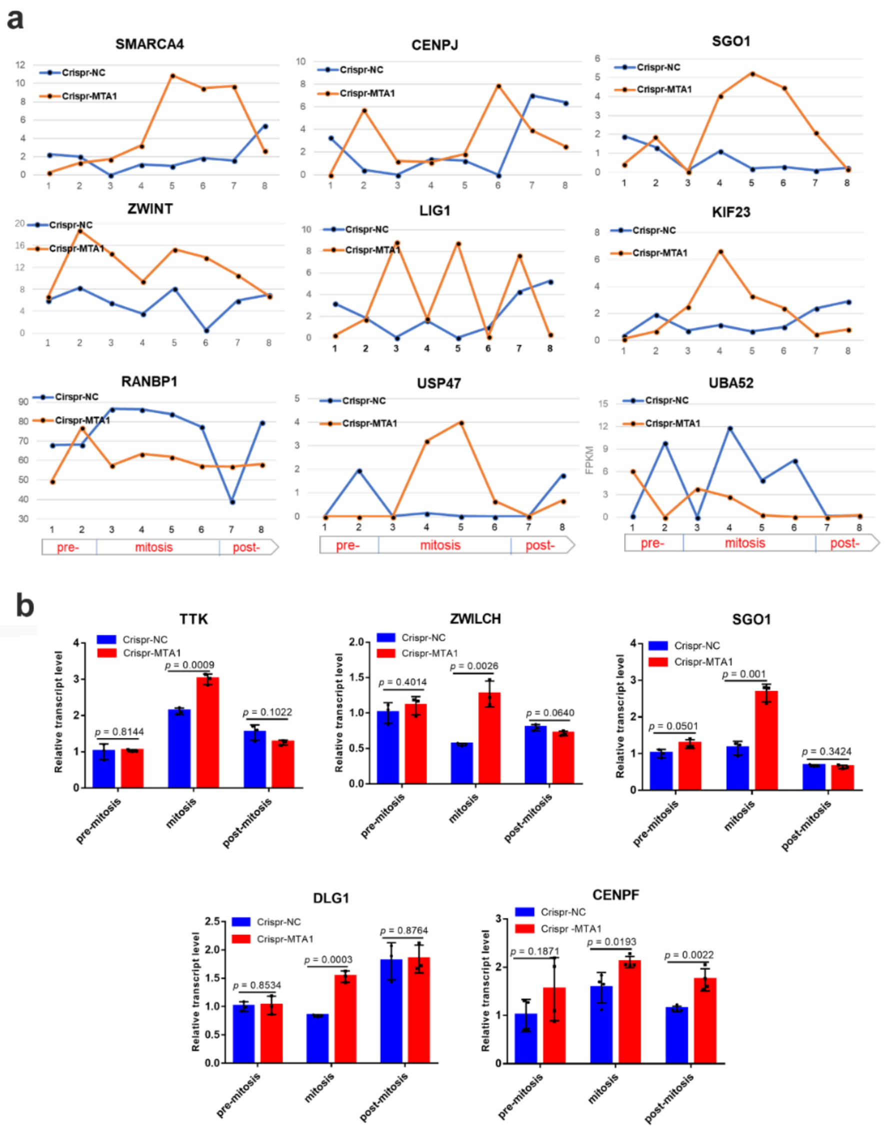

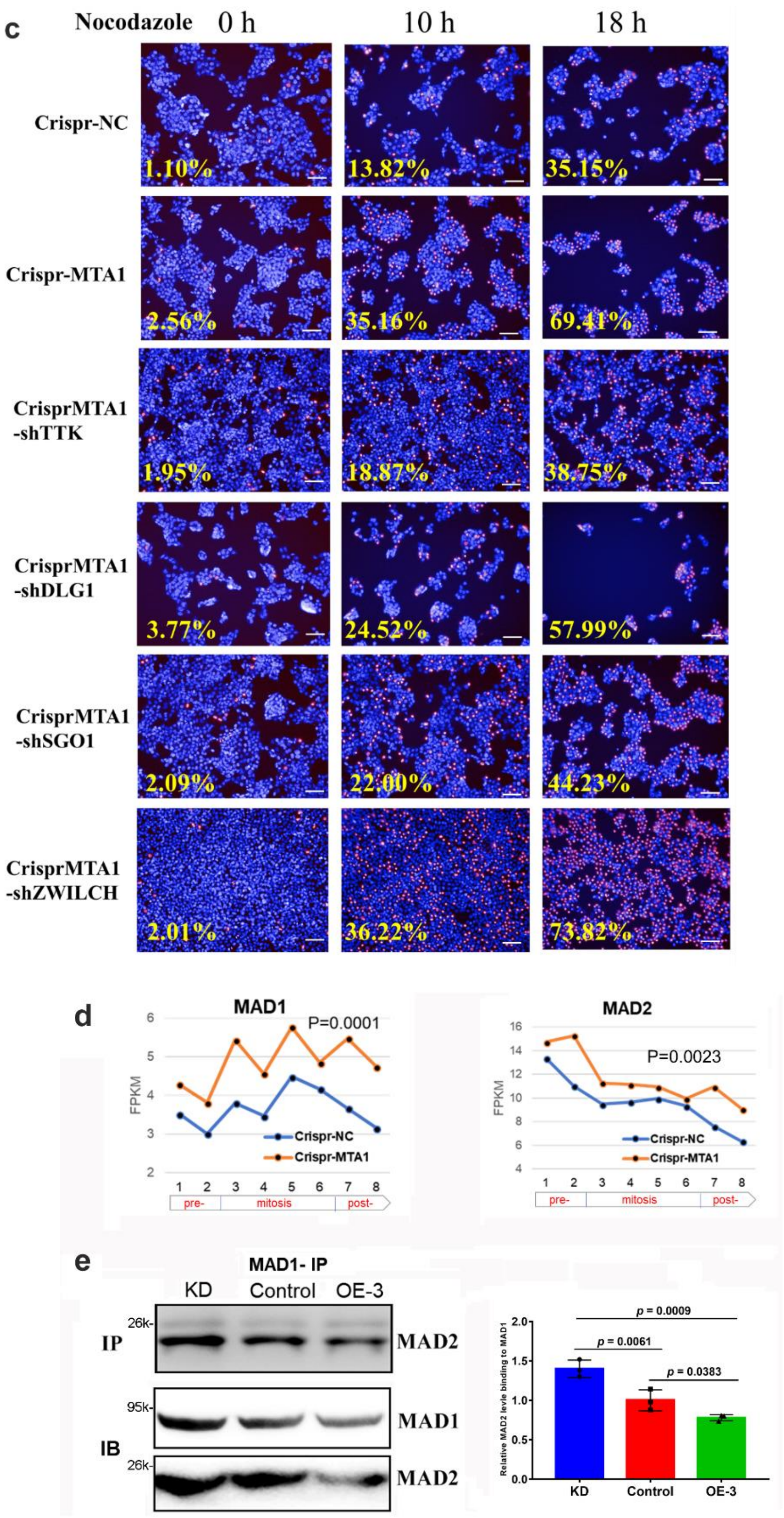

Supplementary Fig. 7 MTA1 alters transcript abundance of mitosis regulators. a MTA1 deletion-caused cell cycle- associated alterations in *SMARCA4*, *CENPJ*, *SGO1*, *ZWINT*, *LIG1*, *KIF23*, *RANBP1*, *USP47* and *UBA52* transcripts. **b** qPCR analysis on

expression of *TTK*, *ZWILCH, SOG1*, *DLG1* and *CENPF* transcripts in pre-mitosis, mitosis and post-mitosis cell samples (n = 3 per group except *CENPF* in which n =4)**.** Two-tailed Student's t test. Data are represented as mean ± SD. **c** Immunofluorescent images captured by high content analysis system showing the mitotic cells labeled by p-H3 after nocodazole treatment. Shown is representative result of three independent repeats with similar results. Scale bar = 100μm. **d** Expression of *MAD1* and *MAD2* mRNA in control and MTA1-knockout HCT116 cells according to the RNA-seq data. Two-tailed paired t test. **e** Co-IP examination of the MAD1-MAD2 interaction using MAD1 antibody in HCT116 cells with different MTA1 expression levels. Data are represented as mean ± SD (n = 3 per group). One-way ANOVA with Holm-Sidak post correction for multiple testing.

Supplementary Figure 8

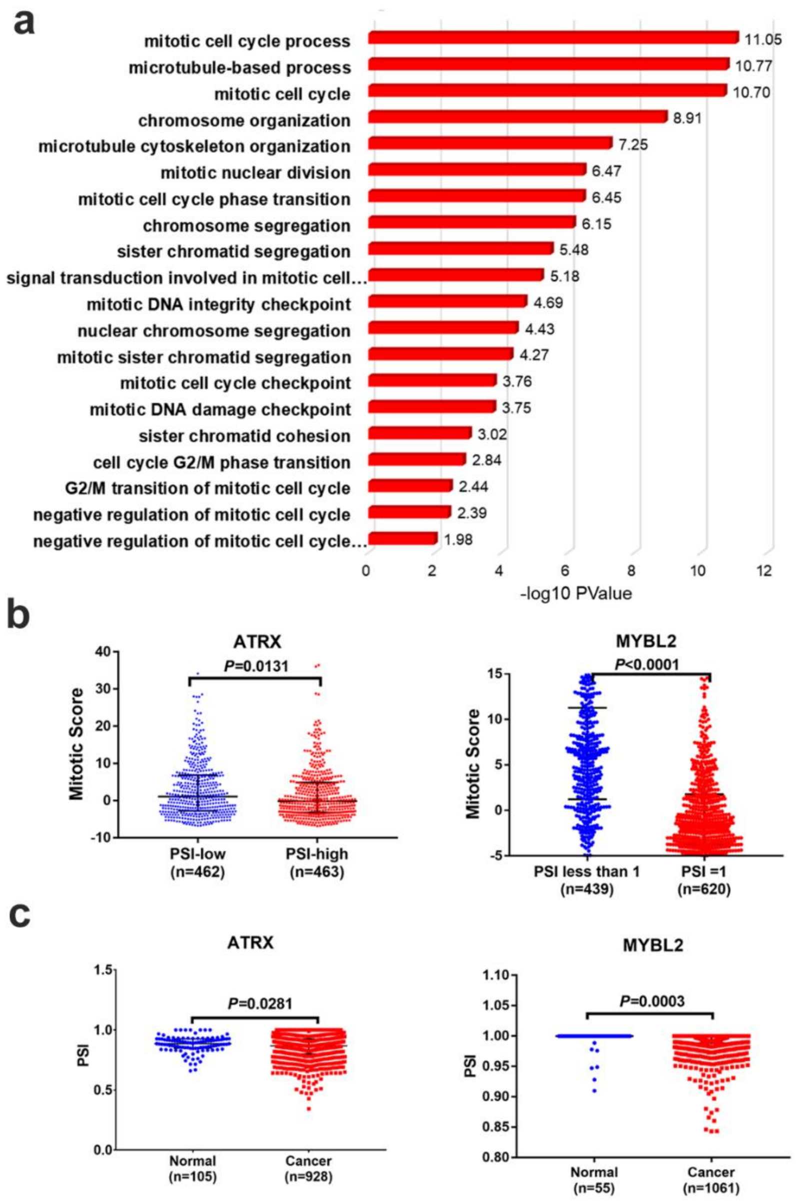

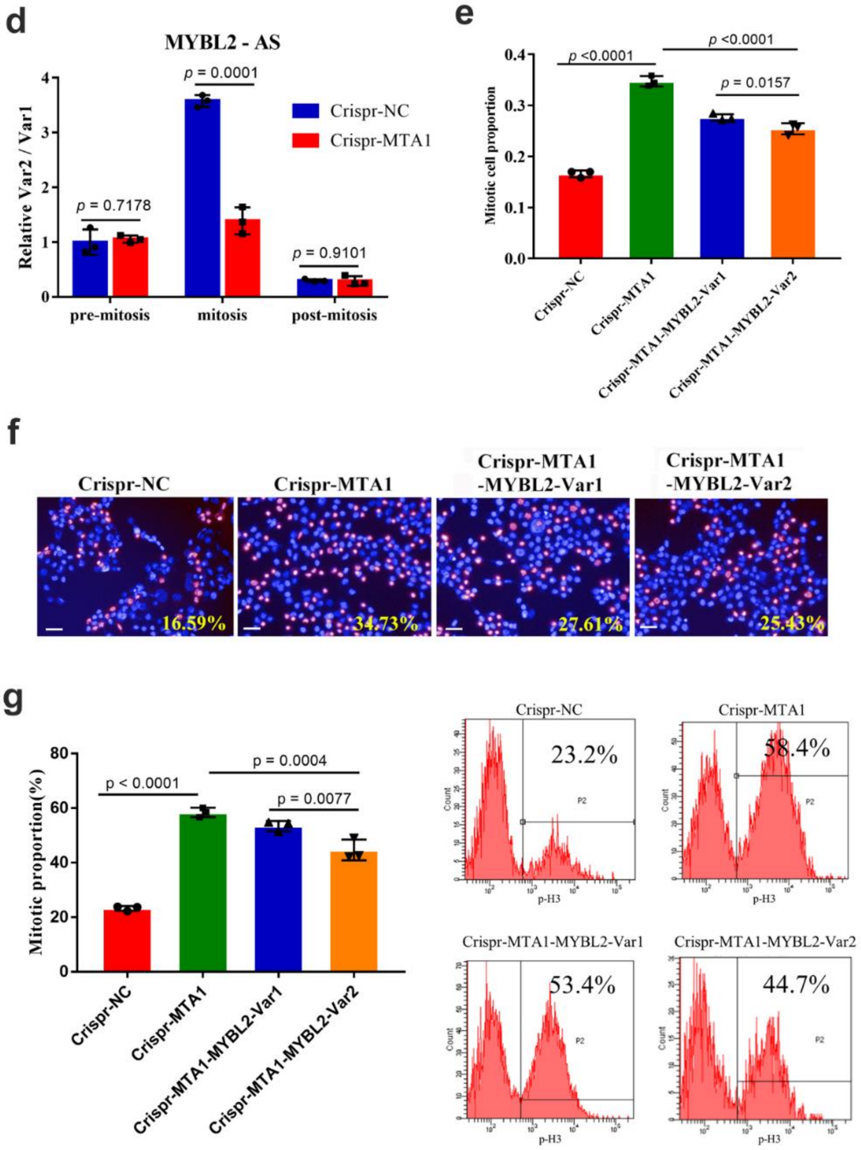
d
MYBL2 - AS
Relative Var2 / Var1
p = 0.7178
p = 0.0001
p = 0.9101
Crispr-NC
Crispr-MTA1
pre-mitosis
mitosis
post-mitosis
e
Mitotic cell proportion
p <0.0001
p <0.0001
p = 0.0157
Crispr-NC
Crispr-MTA1
Crispr-MTA1-MYBL2-Var1
Crispr-MTA1-MYBL2-Var2
f
Crispr-NC
Crispr-MTA1
Crispr-MTA1 -MYBL2-Var1
Crispr-MTA1 -MYBL2-Var2
16.59%
34.73%
27.61%
25.43%
g
Mitotic proportion(%)
p < 0.0001
p = 0.0004
p = 0.0077
Crispr-NC
Crispr-MTA1
Crispr-MTA1-MYBL2-Var1
Crispr-MTA1-MYBL2-Var2
Crispr-NC
23.2%
Crispr-MTA1
58.4%
Crispr-MTA1-MYBL2-Var1
53.4%
Crispr-MTA1-MYBL2-Var2
44.7%
Count
p-H3
P2

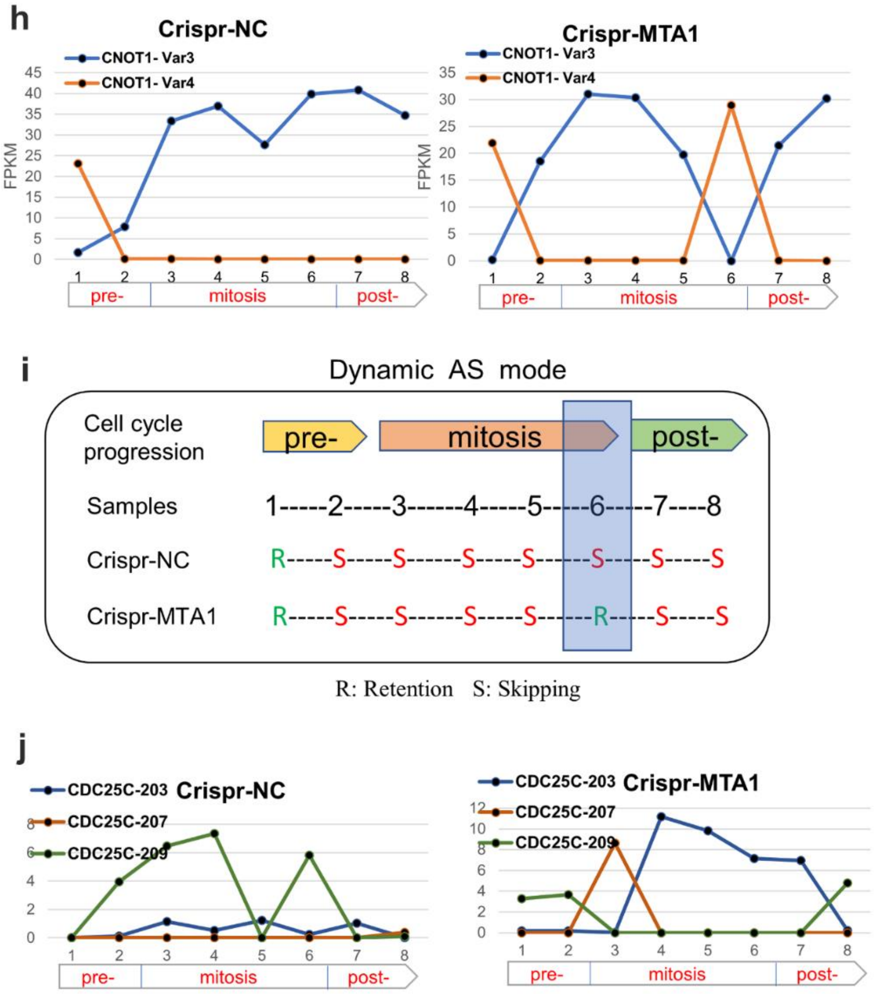

Supplementary Fig. 8 MTA1 regulates AS of mitosis regulators. a MTA1 deletion induced-RASGs show a significant enrichment on the mitosis-related processes as indicated by the functional clustering analysis using DAVID. Modified one-tailed Fisher's exact test was used to calculate p values by DAVID. Only the mitosis-related items were shown. **b** Correlation analysis between the AS events of *ATRX* and *MYBL2* with mitotic score in the TCGA breast cancer dataset. Mitotic score was defined using the sum of expression values for multiple mitosis regulators (CCNB1, CCNB2, CENPF, KIF2C and AURKA). **c** Correlation analysis between the AS events of *ATRX* and *MYBL2* with tumorigenesis in TCGA breast cancer dataset. **d** qPCR analysis of the *MYBL2* var2 / var1 ratio in pre-mitosis, mitosis and post-mitosis samples of control and MTA1-knockout cells (n=3 each). **e-f** High content analysis measuring the nocodazole-induced mitotic arrest proportion in control, MTA1-knockout, MTA1-knockout with MYBL2-Var1 overexpression

and MTA1-knockout with MYBL2-Var2-overexpressing HCT116 cells (n = 3 each). Bar graphs represent mean ± SD. Unpaired two-tailed Student’s t test. Scale bar = 50μm. **g** Flow cytometry analysis examining the nocodazole-induced mitotic arrest proportion in control, MTA1-knockout, MTA1-knockout with MYBL2-Var1 overexpression and MTA1-knockout with MYBL2-Var2 overexpression HCT116 cells (n = 3 each). **h-i** RNA-seq data showing the specific AS pattern conversion from exon skipping to retention at the time point before the mitotic exist after MTA1 deletion. **j** In the control cells, the variant *CDC25C-203* was the dominant isoform across the cell cycle, while in MTA1 deleted cells, the dominate isoforms were changed to *CDC*25C-207 and *CDC25C-209*, depending on the cell cycle stage. **b-c** Two-tailed Mann-Whitney test. Data represents median with interquartile range. **d** Two-tailed Student’s t test. Data represents mean ± SD. **e** One-way ANOVA with Holm-Sidak’s multiple comparisons test. Data represents mean ± SD. **g** One-way ANOVA with Tukey’s multiple comparisons test. Data represents mean ± SD. PSI, the Percent Spliced In value. R: exon retention; S: exon skipping.

Supplementary Figure 9

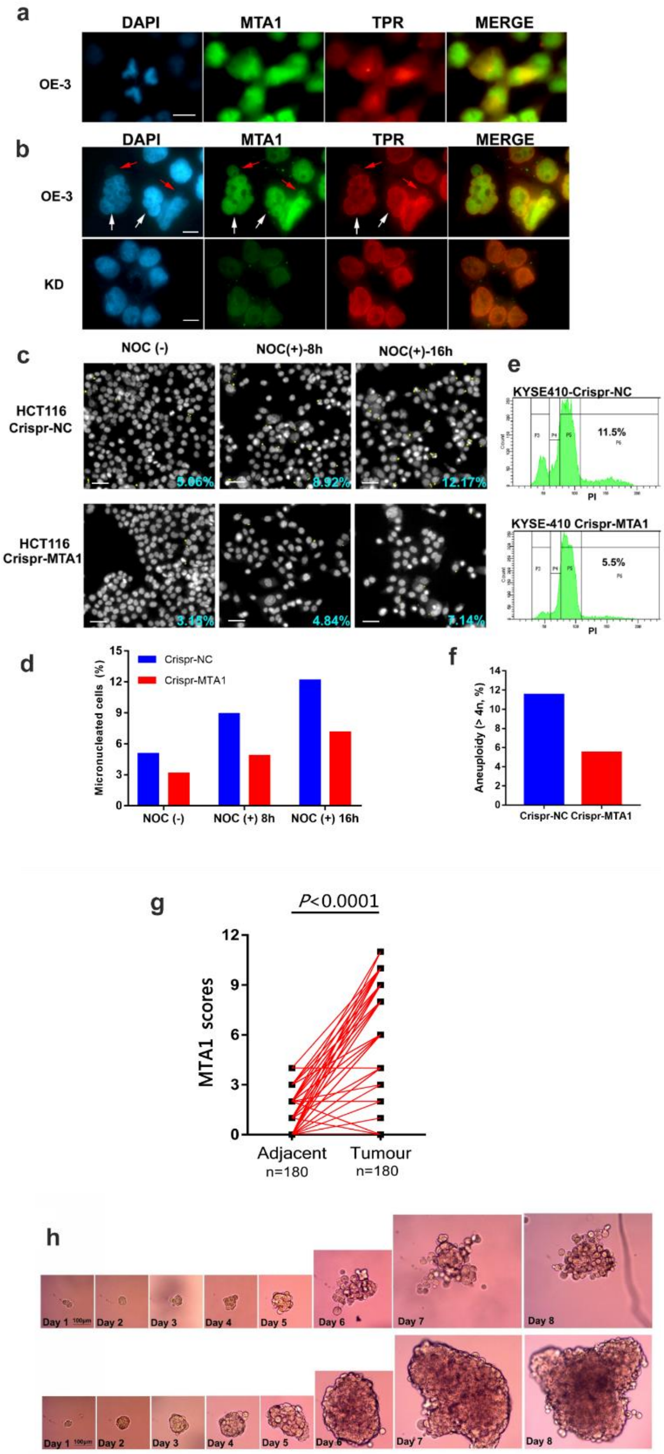

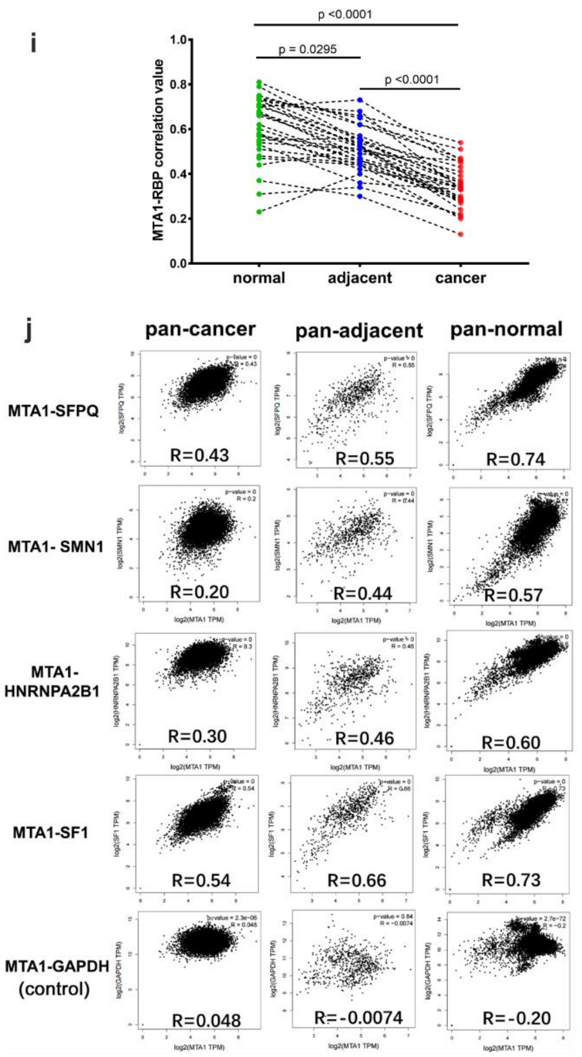

Supplementary Fig. 9 MTA1 induces defective mitosis and CIN occurrence. a Representative images showing the multipolarized chromosomes in MTA1 stable overexpressing HCT116 cells. Blue, DAPI; Green, MTA1; Red, TRP; **b** Representative images showing the occurrence of multinucleated (white arrow) and micronucleated (red arrow) cells in MTA1-overexpression or -deletion HCT116 cell lines. Blue, DAPI; Green, MTA1; Red, TRP. **c-d** High content analyses to detect the spontaneous or nocodazole-induced micronuclei in the control and MTA1 deletion cell lines. The micronuclei are marked with yellow**. e-f** Flow cytometry analyses of the cells with aneuploidy (>4n) in the MTA1-deleted and control KYSE410 cancer cells**. g** Comparison of MTA1 expression in the tumor and paired adjacent tissues. Paired t test, two-tailed. **h** Representative images showing the growth of single cell-derived tumor sphere in control or MTA1 overexpression HCT116 cells from day 1 to day 8. **i** Pathological perturbations in the mRNA coexpression

of MTA1 with splicing factors (14 from our MTA1-interactome and the other 16 were randomly selected from the literature) in GTEx pan-normal, TCGA pan-adjacent and TCGA pan-cancer tissues. Nonparametric Friedman test with Dunn's multiple comparisons test. **j** Correlations of MTA1 with representative interacted splicing factors in normal (GTEx), adjacent (TCGA) and cancer (TCGA) tissues. Pearson correlation analysis by GEPIA (http://gepia.cancer-pku.cn/). All p values < 0.0001 for *MTA1*-RBPs correlation, while for *MTA1-GAPDH* correlation, p value =2.3e-06 (pan-caner), 0.84 (pan-adjacent) and 2.73e-72 (pan-normal), respectively. **a, b** Shown are representative of three independent repeats with similar results. Scale bar = 10 μm for **a** and **b**, 50 μm for **c** and 100 μm for **h**.

Supplementary Figure 10

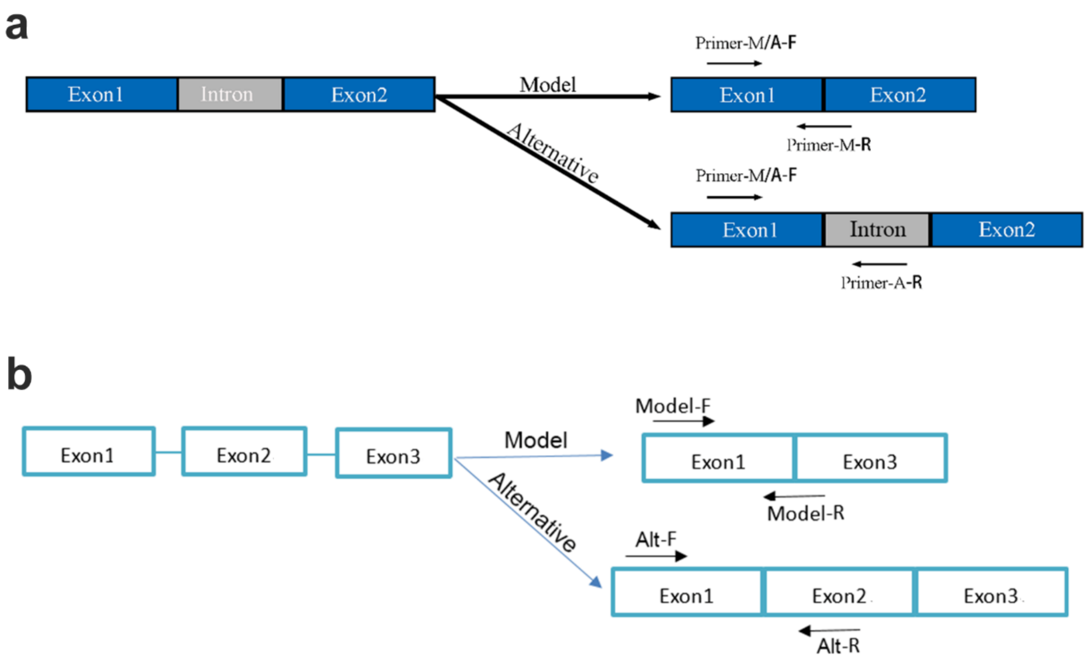

Supplementary Fig. 10 Designs for qRT-PCR validation of ASEs. a The primer design for IR validation. **b** The primer design for the Cassette Exon events validation

Supplementary Table 1

Supplementary Table 1. Correlation between MTA1 level and pathological mitosis.

**Pathologic mitotic cells**	**MTA1 intensity**				**total**
	**Negative**	**Low**	**Moderate**	**High**	
Negative	10（90.91%）	6（50.00%）	17（26.56%）	6（6.45%）	39
Positive	1（9.09%）	6（50.00%）	47（73.44%）	87（93.55%）	141
total	11	12	64	93	180

Supplementary Table 2

Supplementary Table 2. MTA1 promotes tumorigenesis in vivo.

**Cell number/injection**	**Tumorigenesis rate**				
	**HCT116-parent**	**HCT116-Control**	**HCT116-OE-3**	**HCT116-OE-2**	**HCT116-OE-1**
**$2*10^6$**	6/6	6/6	6/6	6/6	6/6
**$1*10^6$**	2/6	1/6	6/6	6/6	6/6
**$5*10^5$**	1/6	2/6	4/6	3/6	5/6
**$1*10^5$**	0/6	0/6	2/6	2/6	1/6

Supplementary Table 3

Supplementary Table 3. Comparison of MTA1 interactome with others from public resources.

Interactome	Antibodies	Number of total captured proteins	Number of captured RBPs	RBP proportion	Reference (PMID)	CRApome accession number
IgG controls	IgG (CC195)	82	15	18.29%	25792328	CC195
	IgG (CC196)	160	45	28.13%	25792328	CC196
	IgG (CC197)	107	26	24.30%	25792328	CC197
	IgG (CC198)	98	21	21.43%	25792328	CC198
	IgG (CC199)	165	42	25.45%	25792328	CC199
	IgG	294	92	31.29%	31501460	-
non-RBPs (proteins without known RNA-binding activity)	anti-MYC	33	11	33.33%	32140074	-
	anti-APE1	444	149	33.56%	31913336	-
	anti-LON	270	24	8.89%	25675302	-
	anti-PAUF	414	55	13.29%	31095674	-
classical RBPs or proteins with RNA-binding activity	anti-hnRNPD	90	58	64.44%	26318153	-
	anti-YBX1	124	89	71.77%	25497084	-
	anti-SOX2	137	94	68.61%	21280222	-
MTA1	anti-MTA1 (mouse)	232	126	54.31%	-	-
	anti-MTA1 (rabbit)	136	69	50.74%	-	-
	anti-MTA1（overlapped）	95	57	60.00%	-	-

Supplementary Table 4

Supplementary Table 4. Quantitative PCR primers and antibodies used.

Gene	Forward (5' to 3')	Reverse (5' to 3')
ITGB4	ATGAGCCGCAACGATGAAC	TTGGTGACAGCAAAGATGGG
EIF4G4	GGAAGAAGGAGAAGCAGGAGA	GAGACAAGTTGGCTGGAATAGG
CD151	GGCGAGACAGTGAGTGGAT	TGTAGATGTTGGAGGCATGGT
CTTN	AAGGTTTCGGCGGCAAATAC	TTCTGGGACTCGTGCTTCTC
MAPK14	GCTCGGCACACAGATGATG	ATCCAGTTCAGCATGATCTCAG
AKT	GCTGGAGGACAATGACTACG	AGCCCTGAAAGCAAGGACTT
RBM15	GGCACCATACGCACCATAGA	ACTGCTGCTGGTAACGATGT
SNRPD1	ACCCTGAAGAACAGAGAACCT	CCACAAGTAGTGTATCCAGAGG
HMGA1	ACTCTTCCACCTGCTCCTTAG	GCTTCTCAGTGCCGTCCTT
TIMP3	TTGTGACTTCCAAGAACGAGTG	GGGTCTGTGGCATTGATGATG
NEAT1-Middle	GAGGAGGCAGCATCTGTAGG	CACATGAGGAGAGGACCAACA
NEAT1-3P	GCATTCTCACCGTCACTCCT	GCAGCAAGCAGTCCACAGA
FBXO5-Model	TGCAGGGGTGTAGGAAACTAGT	AGCCCCAGCGCAGTGACAG
FBXO5-Alt	TGCAGGGGTGTAGGAAACTAGT	TGCGTGATAGACCCTCCACA
PTBP2-Model	CAAAGTCTGTTTACCCTCTTC	AGTTGTGATTGGTTTCCATC
PTBP2-Alt	TAAATGAAGAGGTGTTTATGGA	AGTTGTGATTGGTTTCCATC
SRSF5-Model	CGTCCGGTAGGAAGTACTAG	CGTCCATATCCCTTGAAGAATC
SRSF5-Alt	CGGAGGATGGCGTCTAATG	CGTCCATATCCCTTGAAGAATC
SLC37A2-Model	CTAGTATGGTCCTAACCCACC	GTTTGCCTTCCCTGCTTG
SLC37A2-Alt	AGAGGCAGCGGGTATAAAGAAA	GCTGGGCTGGCTAATGTTCAA
NFKB1-Model	CGGCGGTCATTTCTCTTCAC	TGGATCATCTTCTGCCATTCTG
NFKB1-Alt	ACACAACGTCCTACATCAAGAA	TGGATCATCTTCTGCCATTCTG
CTTN-Model	ACTATGTGAAAGGGTTTGGA	TCCGACTGAACACCGAATT
CTTN-Alt	ACTATGTGAAAGGGTTTGGA	TTGGAGTAGTCTTTTTGGGA
NLRP1-Model	TGCAGGTCTAGCTCCTTCAG	CAATGTGCTCACGGATGCT
NLRP1-Alt	TGCAGGTCTAGCTCCTTCAG	GGTTACCGCAAGCCAGGAA

RBM26-Model	CACAGGTAGAGTTCTAGCATAT	ATGAAGAAGAATTTCAGGAAGA
RBM26-Alt	CACAGGTAGAGTTCTAGCATAT	GTATTCAACTCTGTTGAGAGAG
NFRKB-Model	GCAGACGCAATACATCCTTACC	AAGCCAGCCACGAGTTCTC
NFRKB-Alt	GCAGACGCAATACATCCTTACC	CCTCATCAGTGCTGCTTCTC
LGALS9-Model	TCCTCTTCGTGCAGTACTTCCA	AATGGGAGCCGGGTTGGCAG
LGALS9-Alt	TCCTCTTCGTGCAGTACTTCCA	ACCCAGCAGGGCTTAGACA
EPOR-Model	AGCAAGGCCGCTTTGCTCTC	GGGCTGTATCATGGACCACC
EPOR-Alt	CCATCCAGGACCCAGTCTAAG	GGGCTGTATCATGGACCACC
TEC-Model	CCTCTGGTTTCTCCTGCCAA	GTGGGAAGTATTCACGGAAGG
TEC-Alt	GCACCACCTCACTAGGCTATA	GTGGGAAGTATTCACGGAAGG
LTK-Model	GGCTGCTCCGAGGCTTGG	AGAGGTTGTCAGTCTCTGAAGCG
LTK-Alt	GGCGGCAGGGGGCGAC	TGGCAGTCTCTCAAAGGGCAGTG
GRB7-Model	GGGCGTGTCATTGAGAAC	TCTCCTCACGGGAAATGC
GRB7-Alt	CTCAGTGCAGCCTGTTCCTG	AGGTCAGTGAAGCGGGTCTG
SLMO1-Model	GCCTGTGCCTGTGCTTGTCA	GCGATCAGTCCAGCCTAATCCT
SLMO1-Alt	ATGCAAAGAAGATGCTTCCCAG	TCAGTCCAGCCTAATCCTCCT
SKA2-Model	CCGGAATTAAGCTCTTCTCTGT	GCCACCGTCTGGTGTCTAA
SKA2-Alt	CCGGAATTAAGCTCTTCTCTGT	CGCATTTGTGCTACTGTGAAA
FGFR1-Model	TCTTCCAGGGCTTCAAGATC	CGTGGAGTTCATGTGTAAGGT
FGFR1-Alt	TCCGCATCCGAGCTATTAATC	CGTGGAGTTCATGTGTAAGGT
FGFR2-Model	CGGCGGCATCTTTCAACA	CCACATGGAGATATGGAAGAGG
FGFR2-Alt	GAGATGGCATCTTCTGGCTC	CCACATGGAGATATGGAAGAGG
TTK	GTGGAGCAGTACCACTAGAAATG	CCCAAGTGAACCGGAAAATGA
DLG1	ATGAAGTGATAGGTCCAGAA	AGGGACAGTGGGAGGAGA
CENPF	CTCTCCCGTCAACAGCGTTC	GTTGTGCATATTCTTGGCTTGC
ZWILCH	CACAGCATCACAAACTGCGAT	CCATTCAGTGACACCAGTCCT
SGO1	AACTCAGCAGTCACCTCATCT	TGCACCTACGTTTAGGCAGAG
MYBL2-Var1	GGATGAGCTGCACTACCA	CAGTCCTGCTGTCCAAACT
MYBL2-Var2	CTGCGAGGATCTGGATGA	CAGTGCGGTTCTCCTCAT

GAPDH	AATCCCATCACCATCTTCCA	TGGACTCCACGACGTACTCA

**Antibody**	**Host**	**Source**	**Catalog number**	**Dilution**
HDAC2 (WB)	mouse	Abcam	Ab51832	1:1000
PTBP1 (WB)	rabbit	Abnova	H00005725-M01	1:1000
OGT (WB)	rabbit	Proteintech	11576-2-AP	1:1000
HNRNPU (WB)	rabbit	Proteintech	16365-1-AP	1:1000
NONO (WB)	rabbit	Proteintech	11058-1-AP	1:1000
STRAP (WB)	rabbit	Proteintech	18277-1-AP	1:1000
YBX1 (WB)	rabbit	Proteintech	20339-1-AP	1:1000
HnRNPA1 (WB)	rabbit	Proteintech	11176-1-AP	1:1000
HnRNPD (WB)	rabbit	Proteintech	12770-1-AP	1:1000
SMN1 (WB)	rabbit	Santa Cruz	sc-15320	1:500
GEMIN7 (WB)	mouse	Santa Cruz	sc-130668	1:500
MTA1 (WB)	rabbit	Abcam	ab71153	1:1000
SFPQ (WB)	rabbit	Proteintech	15585-1-AP	1:1000
DHX9 (WB)	rabbit	Proteintech	17721-1-AP	1:1000
GEMIN3 (WB)	rabbit	Santa Cruz	sc-50405	1:500
GEMIN4 (WB)	mouse	Santa Cruz	sc-166418	1:500
MAD1 (WB)	mouse	Santa Cruz	sc-47746	1:500
MAD2 (WB)	mouse	Santa Cruz	sc-47747	1:500
HnRNPH (WB)	mouse	Santa Cruz	sc-32310	1:500
HnRNPA2 (WB)	mouse	Santa Cruz	sc-32316	1:500
HnRNPA1 (WB)	mouse	Santa Cruz	sc-32301	1:500
Flag (WB)	mouse	Sigma-Aldrich	F3165	1:1000
GAPDH (WB)	rabbit	Cell Signaling Technology	#5174	1:2000
IgG (WB)	rabbit	Zhongshan Golden Bridge Bio-technology	ZB-2301	1:5000

IgG (WB)	mouse	Zhongshan Golden Bridge Bio-technology	ZB-2305	1:5000
MTA1 (IF)	rabbit	Abcam	ab50263	1:100
SMN1 (IF)	rabbit	Santa Cruz	sc-15320	1:100
YBX1 (IF)	rabbit	Proteintech	20339-1-AP	1:100
TPR (IF)	rabbit	Santa Cruz	sc-67116	1:100
MTA1 (IHC)	rabbit	Abcam	ab71153	1:200
IgG/FITC (IF)	rabbit	Zhongshan Golden Bridge Bio-technology	ZF-0311	1:100
IgG/TRITC (IF)	rabbit	Zhongshan Golden Bridge Bio-technology	ZF-0316	1:100
IgG/FITC (IF)	mouse	Zhongshan Golden Bridge Bio-technology	ZF-0312	1:100
IgG/TRITC (IF)	mouse	Zhongshan Golden Bridge Bio-technology	ZF-0313	1:100
MTA1 (IP)	rabbit	Abcam	ab71153	-
MTA1 (IP)	mouse	Abcam	ab50263	-
MTA1 (RIP, ChIP, fCLIP)	rabbit	Cell Signaling Technology	# 5646	-
MAD1 (IP)	mouse	Santa Cruz	sc-47746	-
IgG (IP)	rabbit	Abcam	ab97095	-
IgG (IP)	mouse	Abcam	ab102458	-
Phospho-H3(Ser10) Alexa Fluor 647 (FCM)	rabbit	Cell Signaling Technology	3458S	1:50

WB, Western Blot; IF, Immunofluorescence; IP, Immunoprecipitation; RIP, RNA immunoprecipitation; ChIP, Chromatin immunoprecipitation; fCLIP, Formaldehyde crosslinking immunoprecipitation; FCM, Flow cytometry
