# Supplementary material for: Chromatin modifier MTA1 regulates mitotic transition and tumorigenesis by orchestrating mitotic mRNA processing
Source: Nat Commun. 2020 Sep 8;11:4455. doi: 10.1038/s41467-020-18259-1 (PMC7479136; doi:10.1038/s41467-020-18259-1)
Supplement: Supplementary file 3 — Description of Additional Supplementary Files [file 41467_2020_18259_MOESM3_ESM.pdf]

Description of Additional Supplementary Files

File Name: Supplementary Data 1
Description: MTA1 interactome defined using two specific antibodies.

File Name: Supplementary Data 2
Description: DEGs screened in MTA1 knockout HCT116 cells by FDR<0.01.

File Name: Supplementary Data 3
Description: Summary and the detail information of the RASEs by MTA1 knockout.

File Name: Supplementary Data 4
Description: GO analysis on top MTA1-correlated genes in a single-cell RNA-seq dataset (GSE51254).

File Name: Supplementary Data 5
Description: Functional clustering analysis on MTA1 deletion induced-DEGs in synchronized mitotic cells.

File Name: Supplementary Data 6
Description: Functional clustering analysis on MTA1 deletion induced-RASGs in synchronized mitotic cells.
