# Supplementary material for: Chromatin modifier MTA1 regulates mitotic transition and tumorigenesis by orchestrating mitotic mRNA processing
Source: Nat Commun. 2020 Sep 8;11:4455. doi: 10.1038/s41467-020-18259-1 (PMC7479136; doi:10.1038/s41467-020-18259-1)
Supplement: Supplementary file 10 — Reporting Summary [file 41467_2020_18259_MOESM10_ESM.pdf]

nature research

Corresponding author(s): Haili Qian

Last updated by author(s): Feb 5, 2020

Reporting Summary

Nature Research wishes to improve the reproducibility of the work that we publish. This form provides structure for consistency and transparency in reporting. For further information on Nature Research policies, see Authors & Referees and the Editorial Policy Checklist.

Statistics

For all statistical analyses, confirm that the following items are present in the figure legend, table legend, main text, or Methods section.

n/a	Confirmed	
☐	☒	The exact sample size (*n*) for each experimental group/condition, given as a discrete number and unit of measurement
☐	☒	A statement on whether measurements were taken from distinct samples or whether the same sample was measured repeatedly
☐	☒	The statistical test(s) used AND whether they are one- or two-sided<br>*Only common tests should be described solely by name; describe more complex techniques in the Methods section.*
☐	☒	A description of all covariates tested
☐	☒	A description of any assumptions or corrections, such as tests of normality and adjustment for multiple comparisons
☐	☒	A full description of the statistical parameters including central tendency (e.g. means) or other basic estimates (e.g. regression coefficient) AND variation (e.g. standard deviation) or associated estimates of uncertainty (e.g. confidence intervals)
☐	☒	For null hypothesis testing, the test statistic (e.g. *F*, *t*, *r*) with confidence intervals, effect sizes, degrees of freedom and *P* value noted<br>*Give P values as exact values whenever suitable.*
☒	☐	For Bayesian analysis, information on the choice of priors and Markov chain Monte Carlo settings
☒	☐	For hierarchical and complex designs, identification of the appropriate level for tests and full reporting of outcomes
☐	☒	Estimates of effect sizes (e.g. Cohen's *d*, Pearson's *r*), indicating how they were calculated

Our web collection on statistics for biologists contains articles on many of the points above.

Software and code

Policy information about availability of computer code

Data collection	Next500 (illumina) and Hiseq X were used for sequencing; LTQ Velos (Thermo Scientific) was used for mass spectrometry; Fluorescence (Olympus) or confocal laser scanning (Leica) microscopy were used for acquiring images; QuantStudio 6 Flex System(ABI) was used for real-time PCR.
Data analysis	Bioworks Browser rev. 3.1(Thermo Electron, San Jose, CA); rmats (v.4.0.1); TopHat2; Hisat2 version 2.1.0; stringtie version 1.3.4; edgeR; ABLas; ABLIRC; Piranha; CIMS; fuzznuc; Homer; Bowtie2; MACS14；bedtools；R version 3.6.0; Cytoscape；GraphPad Prism version 7; Cutadapt version 1.7.1 and FASTX Toolkit Version 0.0.13

For manuscripts utilizing custom algorithms or software that are central to the research but not yet described in published literature, software must be made available to editors/reviewers. We strongly encourage code deposition in a community repository (e.g. GitHub). See the Nature Research guidelines for submitting code & software for further information.

Data

Policy information about availability of data

All manuscripts must include a data availability statement. This statement should provide the following information, where applicable:

- Accession codes, unique identifiers, or web links for publicly available datasets
- A list of figures that have associated raw data
- A description of any restrictions on data availability

The fCLIP-seq, ChIP-seq, non-synchronized and synchronized RNA-seq data reported here are available under GEO Super Series GSE123959. The mass spectrometry proteomics data have been deposited to the ProteomeXchange Consortium (http://proteomecentral.proteomexchange.org) via the iProX partner repository with the dataset identifier PXD018242. The source data underlying Fig. 1a, d, e, Fig. 2b, d, h, i, Fig. 3b, h, j, Fig. 4a, c, e, Fig. 5b, c, Fig. 6c, g, Fig. 7b, d, f, h, j and Supplementary Fig. 1a–e, g, n, Fig. 2c–e, Fig. 3a, b, e, f, h, j, Fig. 4a-c, Fig. 5a–b, Fig. 6, Fig.7a, b, d, e, Fig.8a-e, g, h, j, Fig.9d, f, g, i are provided as a Source Data file. All the other data supporting the findings of this study are available within the article and its supplementary information files and from the corresponding author upon reasonable request. The public databases used in this study: Database for Annotation, Visualization and Integrated Discovery 6.8 (DAVID 6.8, https://

david.ncifcrf.gov/); Gene Ontology (GO, http://www.geneontology.org/); The Cancer Genome Atlas (TCGA, https://www.cancer.gov/about-nci/organization/ccg/research/structural-genomics/tcga); Gene Expression Omnibus (GEO, https://www.ncbi.nlm.nih.gov/geo/); Cancer Cell Line Encyclopedia (CCLE, https://portals.broadinstitute.org/ccle); Encyclopedia of DNA Elements (ENCODE, https://www.encodeproject.org/); The contaminant repository for affinity purification (CRAPome, http://www.crapome.org/)

Field-specific reporting

Please select the one below that is the best fit for your research. If you are not sure, read the appropriate sections before making your selection.

☒ Life sciences ☐ Behavioural & social sciences ☐ Ecological, evolutionary & environmental sciences

For a reference copy of the document with all sections, see nature.com/documents/nr-reporting-summary-flat.pdf

Life sciences study design

All studies must disclose on these points even when the disclosure is negative.

Sample size	The sample size was determined based on based on experience from previous experiments.
Data exclusions	Invalid values in the RIP-qPCR assays with no amplification curves were excluded.
Replication	Experiments were repeated two to three times with reproducible results.
Randomization	The mice were randomly allocated into different experimental groups. In vitro samples were randomly allocated into different treatment groups.
Blinding	The investigators were blinded to group allocation during data collection and analysis

Reporting for specific materials, systems and methods

We require information from authors about some types of materials, experimental systems and methods used in many studies. Here, indicate whether each material, system or method listed is relevant to your study. If you are not sure if a list item applies to your research, read the appropriate section before selecting a response.

Materials & experimental systems

n/a	Involved in the study	
☐	☒	Antibodies
☐	☒	Eukaryotic cell lines
☒	☐	Palaeontology
☐	☒	Animals and other organisms
☐	☒	Human research participants
☒	☐	Clinical data

Methods

n/a	Involved in the study	
☐	☒	ChIP-seq
☐	☒	Flow cytometry
☒	☐	MRI-based neuroimaging

Antibodies

Antibodies used

Antibody Host Source Catalog number Dilution
HDAC2 (WB) mouse Abcam Ab51832 1:1000
PTBP1 (WB) rabbit Abnova H00005725-M01 1:1000
OGT (WB) rabbit Proteintech 11576-2-AP 1:1000
HNRNPU (WB) rabbit Proteintech 16365-1-AP 1:1000
NONO (WB) rabbit Proteintech 11058-1-AP 1:1000
STRAP (WB) rabbit Proteintech 18277-1-AP 1:1000
YBX1 (WB) rabbit Proteintech 20339-1-AP 1:1000
HnRNPA1 (WB) rabbit Proteintech 11176-1-AP 1:1000
HnRNPD (WB) rabbit Proteintech 12770-1-AP 1:1000
SMN1 (WB) rabbit Santa Cruz sc-15320 1:500
GEMIN7 (WB) mouse Santa Cruz sc-130668 1:500
MTA1 (WB) rabbit Abcam ab71153 1:1000
SFPQ (WB) rabbit Proteintech 15585-1-AP 1:1000
DHX9 (WB) rabbit Proteintech 17721-1-AP 1:1000
GEMIN3 (WB) rabbit Santa Cruz sc-50405 1:500
GEMIN4 (WB) mouse Santa Cruz sc-166418 1:500
MAD1 (WB) mouse Santa Cruz sc-47746 1:500
MAD2 (WB) mouse Santa Cruz sc-47747 1:500
HnRNPH (WB) mouse Santa Cruz sc-32310 1:500

	HnRNPA2 (WB) mouse Santa Cruz sc-32316 1:500<br>HnRNPA1 (WB) mouse Santa Cruz sc-32301 1:500<br>Flag (WB) mouse Sigma-Aldrich F3165 1:1000<br>GAPDH (WB) rabbit Cell Signaling Technology #5174 1:2000<br>IgG (WB) rabbit Zhongshan Golden Bridge Bio-technology ZB-2301 1:5000<br>IgG (WB) mouse Zhongshan Golden Bridge Bio-technology ZB-2305 1:5000<br>MTA1 (IF) rabbit Abcam ab50263 1:100<br>SMN1 (IF) rabbit Santa Cruz sc-15320 1:100<br>YBX1 (IF) rabbit Proteintech 20339-1-AP 1:100<br>TPR (IF) rabbit Santa Cruz sc-67116 1:100<br>MTA1 (IHC) rabbit Abcam ab71153 1:200<br>IgG/FITC (IF) rabbit Zhongshan Golden Bridge Bio-technology ZF-0311 1:100<br>IgG/TRITC (IF) rabbit Zhongshan Golden Bridge Bio-technology ZF-0316 1:100<br>IgG/FITC (IF) mouse Zhongshan Golden Bridge Bio-technology ZF-0312 1:100<br>IgG/TRITC (IF) mouse Zhongshan Golden Bridge Bio-technology ZF-0313 1:100<br>MTA1 (IP) rabbit Abcam ab71153 -<br>MTA1 (IP) mouse Abcam ab50263 -<br>MTA1 (RIP, ChIP, fCLIP) rabbit Cell Signaling Technology # 5646 -<br>MAD1 (IP) mouse Santa Cruz sc-47746 -<br>IgG (IP) rabbit Abcam ab97095 -<br>IgG (IP) mouse Abcam ab102458 -<br>Phospho-H3(Ser10) Alexa Fluor 647 (FCM) rabbit Cell Signaling Technology 3458S 1:50
Validation	All antibodies were purchased from commercial companies and have been validated by the companies.

Eukaryotic cell lines

Policy information about cell lines

Cell line source(s)	The HCT116 and HEK293T cell lines were obtained from the National Infrastructure of Cell Line Resources (Beijing, China). KYSE410 was a kind gift from Dr. Takayoshi Tobe at Kyoto University.
Authentication	The cell lines were authenticated by STR profiling.
Mycoplasma contamination	The cell lines were not mycoplasma-contaminated.
Commonly misidentified lines<br>(See ICLAC register)	There are no misidentified cell lines used in this article

Animals and other organisms

Policy information about studies involving animals; ARRIVE guidelines recommended for reporting animal research

Laboratory animals	For the subcutaneous xenograft experiments, female NU/NU nude mice, at 5-6 weeks of age, were purchased from the Beijing Vital River Laboratory Animal Technology Co., Ltd.
Wild animals	No wild animals were used in this study.
Field-collected samples	No field-collected samples were used in this study.
Ethics oversight	The experimental protocols performed on the animals were approved by The Institutional Animal Care and Use Committee of Cancer Hospital, Chinese Academy of Medical Sciences & Peking Union Medical College.

Note that full information on the approval of the study protocol must also be provided in the manuscript.

Human research participants

Policy information about studies involving human research participants

Population characteristics	Colorectal cancer patients were recruited from the National Cancer Center/National Clinical Research Center for Cancer. Patients ranged in age from 27 to 87 and included both male and female patients. As for the genotypic information, the patients included 164 cases with microsatellite instability and 16 cases with microsatellite stability. The primary treatment was oxaliplatin, and some combined with fluorouracil, xeloda, or cetuximab and so on.
Recruitment	Patients were recruited from the colorectal surgery of National Cancer Center/National Clinical Research Center for Cancer. The consent process required that they were informed by a physician that their participation is not intended to benefit them directly but to help future patients with cancer and that they may withdraw consent at any time. All patients, pre- treatment, treatment, and follow-up information was collected and tracked prospectively for subsequent analysis. There was no inclusion or exclusion criteria for recruitment thus has no potential self-selection bias.
Ethics oversight	The study protocol was approved by the Ethics Committee of Cancer Hospital, Chinese Academy of Medical Sciences & Peking

Ethics oversight | Union Medical College.

Note that full information on the approval of the study protocol must also be provided in the manuscript.

ChIP-seq

Data deposition

☒ Confirm that both raw and final processed data have been deposited in a public database such as GEO.

☒ Confirm that you have deposited or provided access to graph files (e.g. BED files) for the called peaks.

Data access links<br>*May remain private before publication.*	https://www.ncbi.nlm.nih.gov/geo/query/acc.cgi?&acc=GSE123959
Files in database submission	Chip-seq_1st_input_raw_end1.fq.gz<br>Chip-seq_1st_input_raw_end2.fq.gz<br>Chip-seq_1st_MTA1_raw_end1.fq.gz<br>Chip-seq_1st_MTA1_raw_end2.fq.gz<br>Chip-seq_2nd_input_raw_end1.fq.gz<br>Chip-seq_2nd_input_raw_end2.fq.gz<br>Chip-seq_2nd_MTA1_raw_end1.fq.gz<br>Chip-seq_2nd_MTA1_raw_end2.fq.gz<br>Chipseq_1st_peaks.bed<br>Chipseq_2nd_peaks.bed
Genome browser session<br>(e.g. UCSC)	https://www.ncbi.nlm.nih.gov/geo/query/acc.cgi?&acc=GSE123959

Methodology

Replicates	Two replicates.
Sequencing depth	For two MTA1_ChIP samples, 32987944 and 24283084 raw reads were obtained by 151nt paired-end format. For two Input control samples, 23916258 and 22711240 raw reads were obtained by 151 paired-end format.
Antibodies	MTA1 (D17G10) Rabbit mAb (lot number #5646) antibody from Cell Signaling Technology, Inc. (USA) was used for ChIP experiment.
Peak calling parameters	bowtie2 -t -N 0 -p 24 -x bowtie2_index/GRCh38.fa -U Ctrl_1st_DNA_clean.fq -S accepted_hits.sam<br>macs14 -t MTA1_1st_mapping/accepted_hits.uniq.bam -c Ctrl_1st_mapping/accepted_hits.uniq.bam -f BAM -n MTA1_1st_vs_Ctrl_1st -g hs –w
Data quality	We used MACS method to call peaks and then filtered the low-quality peaks. We obtained 7308 and 32042 peaks for the 1st and 2nd replicates, respectively, with p-value 1e-5 and 5-fold enrichment criteria.
Software	The ChIP-seq libraries were sequenced on an Illumina Next500 for pair-end reads, following the manufacturer's instructions. After obtaining the raw reads from the sequencing platform, we used Cutadapt (version 1.7.1) and FASTX Toolkit (Version 0.0.13) to remove adapters and low quality bases (30% bases quality less than 20). The clean reads were mapped to the genome by Botwie2 with no more than 1 mismatch. Uniquely aligned reads were used to identify the MTA1 binding sites by MAC14 with default parameters. All the peaks in each sample were clustered by bedtools (peaks with at least 1 bp overlap were merged together).

Flow Cytometry

Plots

Confirm that:

☒ The axis labels state the marker and fluorochrome used (e.g. CD4-FITC).

☒ The axis scales are clearly visible. Include numbers along axes only for bottom left plot of group (a 'group' is an analysis of identical markers).

☒ All plots are contour plots with outliers or pseudocolor plots.

☒ A numerical value for number of cells or percentage (with statistics) is provided.

Methodology

Sample preparation	Cells from the experimental group and the control group were collected by trypsinization, followed by washing three times with PBS and then fixed with 70% ethanol at 4°C overnight. The next day, centrifuging at 1000 rpm for two minutes, and then adding staining buffer and indicated markers.

Instrument	BD LSRII
Software	FACS DIVA
Cell population abundance	The number of total cells in the tube was 500,000 to 1 million, and the fluorescence of randomly selected 10,000 cells were detected by flow cytometry instrument.
Gating strategy	Propidium Iodide (PI) was used to stain the nuclear DNA, and the G2/M cells were marked with 4N DNA content. The mitotic cells were marked by antibody against mitosis-specific phosphorylated Histone 3.

☒ Tick this box to confirm that a figure exemplifying the gating strategy is provided in the Supplementary Information.
